# Novel biallelic mutations in *TTC29* cause asthenoteratospermia and male infertility

**DOI:** 10.1002/mgg3.2078

**Published:** 2022-11-08

**Authors:** Siyu Dai, Yan Liang, Mohan Liu, Yanting Yang, Hongqian Liu, Ying Shen

**Affiliations:** ^1^ Core Facility West China Hospital, Sichuan University Chengdu China; ^2^ Department of Obstetrics and Gynecology West China Second University Hospital, Sichuan University Chengdu China; ^3^ Medical Genetics Department, Prenatal Diagnostic Center West China Second University Hospital, Sichuan University Chengdu China; ^4^ Key Laboratory of Birth Defects and Related Diseases of Women and Children, Ministry of Education Sichuan University Chengdu China; ^5^ State Key Laboratory of Biotherapy and Cancer Center Sichuan University Chengdu China; ^6^ Department of Obstetrics/Gynecology, Joint Laboratory of Reproductive Medicine (SCU‐CUHK), Key Laboratory of Obstetric, Gynecologic and Pediatric Diseases and Birth Defects of Ministry of Education West China Second University Hospital, Sichuan University Chengdu China

**Keywords:** asthenoteratozoospermia, gene mutations, male infertility, MMAF, *TTC29*

## Abstract

**Background:**

Multiple morphological abnormalities of the sperm flagella (MMAF), which is characterized as asthenoteratospermia involving absent, short, bent, coiled, and/or irregular‐caliber flagella, is a rare recessive inherited disorder associated with male infertility. To date, genetic causes of MMAF cases are not fully explored.

**Methods:**

Whole‐exome sequencing was conducted to identify pathogenic variants in a patient with MMAF. The functional effect of the identified mutations was investigated by immunofluorescence staining and western blotting. Intracytoplasmic sperm injection was used to assist fertilization for the patient with MMAF.

**Results:**

We identified novel biallelic mutations, a splicing variant NC_000004.12:g.146937593C>T (c.254+1G>A), and a nonsense mutation NM_001300761.4:c.1185C>G (NP_001287690.1:p.Tyr395*), in *TTC29* from an infertile patient. In addition to the typical MMAF phenotype, the patient also presented aberrant morphology of sperm heads. Further functional experiments confirmed the absence of TTC29 expression in the spermatozoa. We also explored the specific expression pattern of *TTC29* in human and mouse spermatogenesis. The outcome of intracytoplasmic sperm injection in the patient was unsuccessful, while additional female risk factors should not be excluded.

**Conclusions:**

Our study revealed the novel biallelic mutations in *TTC29* in a MMAF patient, which findings expand the mutational spectrum of *TTC29* and further contribute to the diagnosis, genetic counseling, and prognosis of male infertility.

## 1 BACKGROUND

The WHO has deemed infertility a global health issue, and male infertility is wholly or partly the cause of infertility in 20%–70% of couples (Agarwal et al., 2015; WHO, 2016). Male infertility is a major reproductive disorder that manifests with a highly heterogeneous clinical phenotype of decreased sperm count and/or quality (Agarwal et al., 2021; Tournaye et al., 2017). Male reproductive impairment is caused by various complex factors and clinical entities. The main causes of male infertility are spermatogenesis defects, ductal obstruction or dysfunction, and hypothalamus‐pituitary axis disturbances (Tournaye et al., 2017). These pathogenic factors usually cause damage to spermatogenesis, presenting as azoospermia or varying degrees of oligozoospermia, teratozoospermia, and asthenozoospermia or combinations thereof (Yang et al., 2021). Asthenoteratospermia is one of the main clinical presentations of male infertility, which is characterized by a decline in sperm counts and defective sperm motility with severe flagellar malformations (Shahrokhi et al., 2020).

Multiple morphological abnormalities of the flagella (MMAF), also called short tails, is a subtype of asthenoteratospermia, representing five abnormal flagellar morphologies, including absent, short, coiled, bent, irregular flagella, or all (Ben Khelifa et al., 2014). The pathogenetic mechanisms of MMAF are largely unknown. However, the genetic etiology of MMAF is explored by an increasing number of studies, indicating that MMAF is a disease of highly heterogeneous genetic origin (Touré et al., 2021; Wang et al., 2020). With the development of next‐generation sequencing, the investigation of MMAF‐associated genes has progressed. To date, 42 MMAF‐associated genes have been reported, including *AK7* (MIM:615364), *AKAP3* (MIM:604689), *AKAP4* (MIM:300185), *ARMC2* (MIM:618424), *WDR19* (MIM:608151), *WDR63*/*DNAI3* (MIM:617968), *CEP135* (MIM:611423), *CFAP43*/*WDR96* (MIM:617558), *CFAP44*/ *WDR52*(MIM:617559), *CFAP47* (MIM:301057), *CFAP53* (MIM:614759), *CFAP58* (MIM:619129), *CFAP61*, *CFAP65* (MIM:614270), *CFAP69* (MIM:617949), *CFAP70* (MIM:618661), *CFAP74*, *CFAP91* (MIM:609910), *CFAP251*/*WDR66* (MIM:618146), *CFAP206*, *CCDC34* (MIM:612324), *CCDC38*, *CCDC39* (MIM:613798), *CCDC40* (MIM:613799), *DNAH1* (MIM: 603332), *DNAH2* (MIM:603333), *DNAH6* (MIM: 603336), *DNAH8* (MIM:603337), *DNAH10* (MIM:605884), *DNAH12* (MIM:603340), *DNAH17* (MIM:610063), *DZIP1* (MIM:608671), *DRC1* (MIM:615288), *FSIP2* (MIM:618153), *ODF2* (MIM:602015), *QRICH2* (MIM:618304), *SPEF2* (MIM:610172), *SPAG6* (MIM:605730), *STK33* (MIM:607670), *LRRC46*, *TTC21A* (MIM: 611430), and *TTC29* (MIM: 618735) (Baccetti et al., 2005; Chen et al., 2021; Cong et al., 2022; He et al., 2020; Li et al., 2021; Li et al., 2022; Liu et al., 2020; Liu et al., 2021; Lu et al., 2021; Ma et al., 2021; Ma et al., 2022; Ni et al., 2020; Sha et al., 2020; Shen et al., 2021; Sironen et al., 2020; Tang et al., 2017; Visser et al., 2011; Wu et al., 2021; Xu et al., 2020; Xu, Tang, et al., 2022; Xu, Yang, et al., 2022; Yin et al., 2022; Zhang et al., 2021; Zhang et al., 2022; Zhu et al., 2022). However, there are still many individuals with MMAF that cannot be causally diagnosed, which indicates that there may be other pathogenic mutations not yet discovered. Therefore, more gene mutations of MMAF need to be identified to further understand the genetic causes and potential molecular mechanisms of this disease and eventually provide appropriate diagnosis and therapeutic schedules for patients with MMAF.

*TTC29* is located on chromosome 4 and contains 14 exons, predicting a 501‐amino‐acid protein. In Leishmania, the absence of TTC29 led to the short sperm flagellum and further reduced sperm motility (Beneke et al., 2019). Work from Patrick Lore's et al. reported five infertile individuals carrying three mutations in *TTC29*, including a homozygous splice‐site variant NC_000004.11:g.147858745C>T (c.176+1G>A), a homozygous frameshift variant NM_031956.3:c.330_334delGGAGG, and a homozygous nonsense variant NM_031956.3:c.750C>A, and showing a typical MMAF phenotype (Lorès et al., 2019). What's more, they found that both *TTC29* loss‐of‐function models of mouse and trypanosome showed reduction in sperm flagellar beating and motility, and a significant increase in more minor morphological defects of the flagellum in mice was observed (Lorès et al., 2019). Another study identified biallelic truncating mutations of *TTC29* in three unrelated cases with MMAF, including a homozygous stopgain mutation NM_031956.3:c.1107C>G, a homozygous frameshift mutation NM_031956.3:c.412_425del, and a homozygous splice‐site mutation NC_000004.11:g.147754957C>A (c.977+1G>T) (Liu et al., 2019). In addition, the deficiency of TTC29 significantly decreased staining of intraflagellar‐transport‐complex‐B‐associated proteins (TTC30A and IFT52) in the patients' spermatozoa by immunofluorescence assays (Liu et al., 2019). Furthermore, the decreased sperm motility, aberrant ultrastructure of flagellar, and male subfertility were observed in the *Ttc29*‐mutated male mice (Liu et al., 2019). However, in view of the limited cases of *TTC29* mutations in humans, more identification of pathogenic mutations in *TTC29* in patients with MMAF is important for the genetic diagnosis of male infertility.

In this study, we reported an infertile male with MMAF and discovered novel biallelic variants of *TTC29* in the patient. Functional studies in vitro revealed that the biallelic variants of *TTC29* gave rise to a lack of TTC29 protein expression. Intriguingly, the obvious aberrant morphology of sperm head and acrosome were observed in the patient besides the typical MMAF phenotype. Our study also investigated the specific expression pattern of TTC29 during spermatogenesis in humans and mice. Our findings provide strong evidence to confirm the causative relationship between *TTC29* variants and MMAF‐associated asthenoteratospermia. Furthermore, the novel mutations we discovered expand the *TTC29* gene's mutation spectrum and provide more information for genetic counseling and diagnosis.

## 2 MATERIALS AND METHODS

### 2.1 Ethical compliance

The study was approved by the Ethical Review Board of West China Second University Hospital, Sichuan University (reference number: 202053). Signed informed consent was obtained from all subjects participating in the study.

### 2.2 Study participants

An infertile patient and his parents were enrolled from the West China Second University Hospital of Sichuan University. Family members of the patient were also recruited. A total of 500 unrelated Han Chinese men who fathered naturally conceived children were recruited for the study. No factors of other diseases associated with infertility (such as androgenic or endocrine abnormalities, cryptorchidism, varicocele, seminal ductal obstruction, testicular trauma, or tumor) were observed in the patient after careful clinical examinations. The patient and normal controls underwent routine semen analyses in accordance with World Health Organization guidelines (WHO, 2010). Peripheral whole blood samples were collected for genetic analyses. The chromosomal karyotypes were normal (46; XY), and no large‐scale deletions were found in the Y chromosome.

### 2.3 Whole‐exome sequencing and Sanger sequencing

Genomic DNA was isolated from peripheral blood samples of the patient using a whole‐blood DNA purification kit (Axygen Scientific). One microgram of genomic DNA was used to enrich the human exome using the Agilent SureSelect Human All Exon V6 Kit (Agilent Technologies). Next‐generation sequencing was subsequently conducted with the Illumina HiSeq X system (Illumina) following the manufacturer's instructions. Reads were mapped to the human genome reference assembly (GRCh37/hg19) by using the Burrows Wheeler Aligner (BWA) software to put the original mapping result into BAM format. Subsequently, duplicates were filtered on Picard (http://broadinstitute.github.io/picard/index.html), and Picard was also used to evaluate the quality of variants. Then, ANNOVAR software was used for functional annotation based on the genome Aggregation database (gnomAD), 1000 Genomes Project, Exome Aggregation Consortium Browser (ExAC).

Sanger sequencing was applied to verify the mutation detected by whole‐exome sequencing in the normal controls, the proband, and his family members. Polymerase chain reaction (PCR) amplification was performed with the ProFlex PCR System (Thermo Fisher Scientific). DNA sequencing of PCR products was conducted on an ABI 377A DNA Sequencer (Applied Biosystems). The primers for PCR were as follows: F1: 5′‐TGATCAACCAACGACCTAACTCT‐3′ and R1: 5′‐TGGAGACAACACACCTTAAAATGA‐3′; F2: 5′‐TAATAGCCTGCTTGCCATC‐3′ and R2: 5′‐TACCTCTGCTCTCCTTCCA‐3′.

### 2.4 Papanicolaou staining

First, semen samples were smeared on slides. Then, slides were air‐dried, fixed with 95% (volume/volume) ethanol for at least 15 min, and sequentially immersed in a graded series of ethanol (50%, 80%, 95%), Harris's hematoxylin, acidic ethanol, G‐6 orange stain, and EA‐50 green stain, as described in the WHO guidelines (WHO, 2010). Finally, the stained semen smears were mounted using ethanol‐soluble mounting media (Thermo Fisher Scientific).

### 2.5 Electron microscopy

The ultrastructural features of the spermatozoa from the infertile patient and normal control were analyzed by scanning electron microscopy (SEM) and transmission electron microscopy (TEM). For the SEM assay, the spermatozoa samples were fixed onto slides of 1 cm diameter using 2.5% glutaraldehyde for 4 h at 4°C. After washing the slides with 1× phosphate buffered saline (PBS) three times and postfixing in 1% osmic acid for 1 h at 4°C, dehydration was performed using 30%, 50%, 75%, 95%, and 100% ethanol sequentially, and the slides were dried using a CO_2_ critical‐point dryer (Eiko HCP‐2, Hitachi). Before examination with a field emission SEM Hitachi S3400, the dried specimens were glued to aluminum stubs and Pt sputter‐coated by an ionic sprayer meter (Eiko E‐1020, Hitachi).

For the TEM assay, samples were fixed in 3% glutaraldehyde, phosphate‐buffered to pH 7.4, and postfixed with 1% OsO_4_. After dehydration, the samples were incubated in propylene oxide followed by embedding in a mixture of Epon 812 and Araldite. Ultrathin sections obtained by an Em UC6 Ultramicrotome (Leica) were collected on TEM nickel grids and analyzed using TEM (TECNAI G2 F20, Philips) at 120 kV.

### 2.6 Western blot analysis

The sperm sample from the patient was lysed by RIPA buffer (P0013C, Beyotime) with protease inhibitor cocktail (B14012, Bimake) for extracting total protein. We used Thermo Scientific BCA (23,230, bicinchonic acid) to generate protein assay details, according to the manufacturer's instructions. Lysates were mixed with SDS Sample loading buffer (P0015, Beyotime) and boiled for 10 min. After denaturation, proteins were separated on 10% sodium dodecyl sulphate–polyacrylamide gels (stock gel: 60 V, 30 min; separated gel: 100 V, 1.5 h) and transferred to a 0.45‐μm pore‐size polyvinylidene difluoride membrane (Millipore) by wet transfer (200 mA, 30 min). The transferred membrane was blocked with 5% skimmed milk in tris buffered saline with tween‐20 for 1 h at room temperature and incubated in primary antibody solution at 4°C overnight. The primary antibodies used in this study were anti‐TTC29 (1:500, HPA061473, Sigma–Aldrich) and anti‐GAPDH (1:5000, ab8245, Abcam). Next, the membrane was washed with 1 × tris‐buffered saline with Tween 20 for three times and incubated with goat anti‐mouse IgG secondary antibody‐HRP (1:5000, 32,230, Thermo Fisher Scientific) in 5% skimmed milk at room temperature for 1.5 h. The membrane was then washed with 1 × tris‐buffered saline with Tween 20 for three times. Finally, immunoblots were developed using Thermo Scientific™ Pierce™ ECL Western Blotting Substrate (TWBKLS0500, Millipore).

### 2.7 Immunofluorescence staining

For spermatozoa staining, the spermatozoa samples or selected germ cells from mice and humans were fixed with 4% paraformaldehyde at 4°C for 30 min, permeabilized with 0.3% Triton X‐100, washed with 1× PBS, and blocked with 5% bovine serum albumin in PBS for 1 h. Subsequently, sperm were incubated overnight at 4°C with the following primary antibodies: rabbit polyclonal anti‐TTC29 (1:25, HPA061473, Sigma‐Aldrich) and anti‐α‐tubulin (1:100, A11126, Thermo Fisher Scientific). After washing three times with 1 × PBS, the samples were incubated with secondary antibodies for 2 h at room temperature or coincubated with peanut agglutinin (PNA, 1:50, RL‐1072‐5, Vector). The secondary antibodies were as follows: Alexa Fluor 488 (1:1000; A32723, Thermo Fisher) and Alexa Fluor 594 (1:1000, 1,927,937, Thermo Fisher). Then, the samples were counterstained with 4,6‐diamidino‐2‐phenylindole (DAPI, Sigma‐Aldrich) to label the nuclei. Images were acquired using a laser scanning confocal microscope (Olympus).

For staining of testicular tissues, samples of human and mouse testes were fixed with 4% paraformaldehyde and Bouin's solution overnight at room temperature, embedded in paraffin, and cut into slices. The samples were sectioned at a thickness of 5 μm. The slices were deparaffinized, boiled in 10 mM citrate buffer (pH 6.0) for 10 min, soaked in 3% H_2_O_2_ for 10 min, washed with 1× PBS, and incubated with the primary antibody anti‐TTC29 (1:25, HPA061473, Sigma‐Aldrich) at 4°C overnight. Next, the slices were washed with 1× PBS three times and incubated with the secondary antibody Alexa Fluor 488 (1:1000; A32723, Thermo Fisher) for 1 h at 25°C. Finally, images were captured with a confocal microscope (Olympus FV3000).

### 2.8 Real‐time PCR

TRIzol reagent (Invitrogen) was used to extract total RNA from mouse tissues, and a RevertAid First‐Strand cDNA Synthesis Kit was used to synthesize cDNA (Thermo Fisher Scientific) in line with the manufacturer's protocol in SimpliAmp™ thermal cycler parameters (A24811) (Thermo Fisher Scientific). Real‐time PCR was performed using SYBR Premix Ex Taq II (TaKaRa) on an iCycler RT–PCR Detection System (Bio‐Rad Laboratories). Each assay was performed in triplicate. Agarose gel electrophoresis was used to analyze the amplified products. The 2^−ΔΔCt^ method was used to normalize the real‐time PCR data. The *Gapdh* gene was used as an internal control. The primers for real‐time PCR were as follows: *Ttc29*‐F 5′‐ATGCTGCGAGATGGGTTCC‐3′; *Ttc29*‐R 5′‐CTTCCAGAGCCGTCCGTGT‐3′; *Gapdh*‐F 5′‐AGGTCGGTGTGAACGGATTTG‐3′; *Gapdh*‐R 5′‐TGTAGACCATGTAGTTGAGGTCA‐3′.

### 2.9 Isolation of human and mouse spermatogenic cells

Spermatogenic cells were obtained through cell diameter/density at unit gravity using the STA‐PUT velocity sedimentation method as previously described (Bellvé, 1993; Bellvé et al., 1977). In brief, testicular single‐cell suspensions were obtained from the seminiferous tubules of adult C57BL/6 male mice (DOSSY). Human testis tissue was obtained from an obstructive azoospermia patient with informed consent. The first step is to incubate the seminiferous tubules in 10 ml 1 × PBS with 90 mg/ml of collagenase (Invitrogen) and agitate the seminiferous tubules continuously for 15 min at 32°C. Then, after allowing the digested product to sediment for 5 min, we removed the supernatant. Next, before incubated for 15 min, the sediment was resuspended in 10 ml of 1 × PBS with 60 mg/ml of trypsin (Sigma‐Aldrich) and 1 mg/ml of DNase (Promega). Additionally, before the large cell clumps were filtered out with 40 mm nylon mesh, the cell suspension was centrifuged at 400 ***g*** for 10 min and then we used 1 × PBS to wash the cell suspension three times. Subsequently, containing 0.5% bovine serum albumin, the HEPES‐buffered RPMI (Gibco) was used to resuspended the obtained single cells. The final spermatogenic cell suspensions were sorted by STA‐PUT apparatus (including two gradient glass chambers, one cell loading chamber, one standard sedimentation chamber, plastic tubing and baffles) velocity sedimentation. Several germ cell populations were collected for subsequent analysis.

## 3 RESULTS

### 3.1 MMAF phenotype identified in a patient with asthenoteratospermia

The patient was a 34‐year‐old male diagnosed with infertility for 6 years with a normal chromosome karyotype (46; XY). Semen analysis was conducted in the source laboratories during routine examination of the individuals according to WHO guidelines (Wang et al., 2014). As shown in Table 1, the percentage of normal sperm in the patient dropped remarkably compared to the normal reference value. Almost all of the patient's spermatozoa had abnormal morphology. Notably, no spermatozoa with progressive motility were observed in the patient.

**TABLE 1:** Semen analysis in the patient

	Patient	References
Sperm volume (ml)	1.9	>=1.5
Sperm concentration(10^6^/ml)	56.5	>=15
Motility (A + B, %)	0	>=40
Vitality (%)	38.0	>=58
Normal spermatozoa (%)	1.0	>=4
Defective spermatozoa (%)	99.0	–
Short flagella (%)	19.5	–
Coiled flagella (%)	21.5	–
Absent flagella (%)	23.4	–
Bent flagella (%)	22.7	–
Abnormal head (%)	86.8	–

**FIGURE 1:**
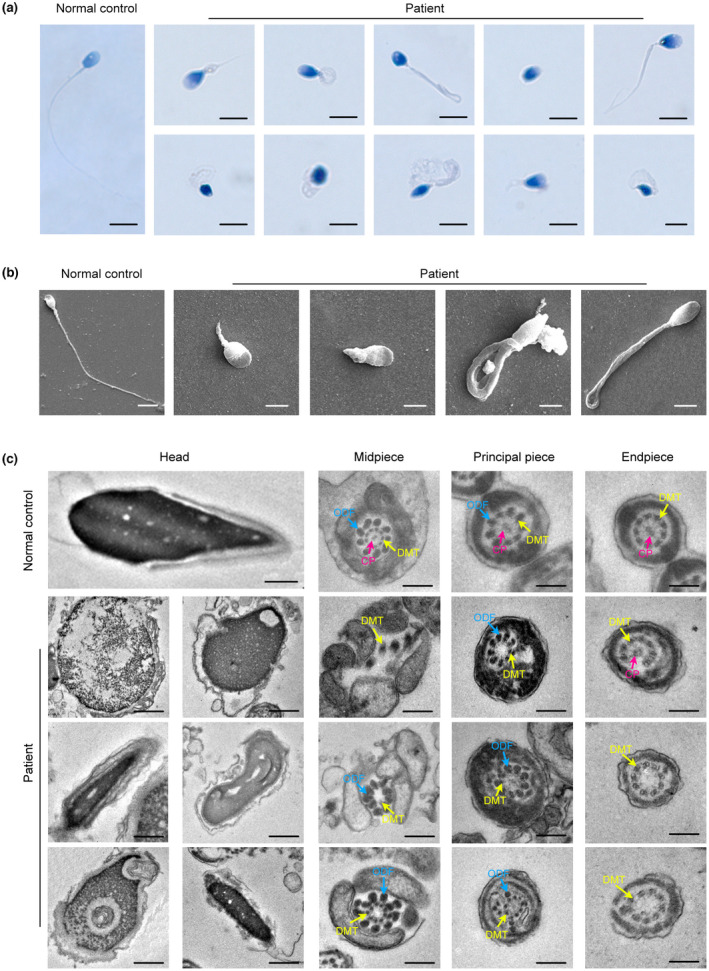
The morphological abnormalities of spermatozoa in the patient. (a) Papanicolaou staining results presented flagellar abnormalities in the patient's spermatozoa and meanwhile showed an aberrant morphology of sperm heads (scale bars, 5 μm). (b) SEM analysis of spermatozoa obtained from a fertile control individual and the patient. Typical phenotype in various shapes of sperm flagella was confirmed by SEM (scale bars, 5 μm). (c) TEM showed the abnormal ultrastructure of the head and flagellum from the patient's spermatozoa compared to the normal control (Scale bars, 200 nm).

Sperm morphology was assessed with Papanicolaou staining and SEM. Typical MMAF phenotype was observed, including absent, short, bent, and coiled flagella (Figure 1a,b and Figure S1). Interestingly, abnormally shaped heads were observed in the patient's spermatozoa, such as round head, small head, pyriform head, amorphous head, and tapered head (Figure 1a). Moreover, various ultrastructural defects were discovered by TEM in the sperm flagella. The typical “9 + 2” microtubule structure was observed in the cross‐sections of the flagella piece from the control individual, including nine double microtubule dipoles (DMT) and a central pair of microtubules (CP) surrounded by nine outer dense fibers (ODF) (Figure 1c). However, a remarkable disorganization in axonemal or peri‐axonemal structures, including disorganized peripheral microtubule doublets and misarranged outer dense fibers with a lack of the central pair of microtubules, were detected in the spermatozoa from the patient compared to the normal control (Figure 1c). Additionally, the nuclei in most sperm from the patient were more unconsolidated, and showed the abnormal vacuoles (Figure 1c). Moreover, most of the acrosomes in the sperm heads from the patient were incomplete and the space between sperm nuclei and acrosomes was widened (Figure 1c). To better understand this phenomenon, PNA staining marking the sperm acrosome was used to analyze defects from the patient's sperm heads. Strikingly, immunofluorescence staining of PNA demonstrated that sperm acrosomes were dramatically defective in the patient compared to the normal control (Figure 3c).

### 3.2 Loss‐of‐function mutations in *TTC29* accounted for MMAF in the patient

**FIGURE 2:**
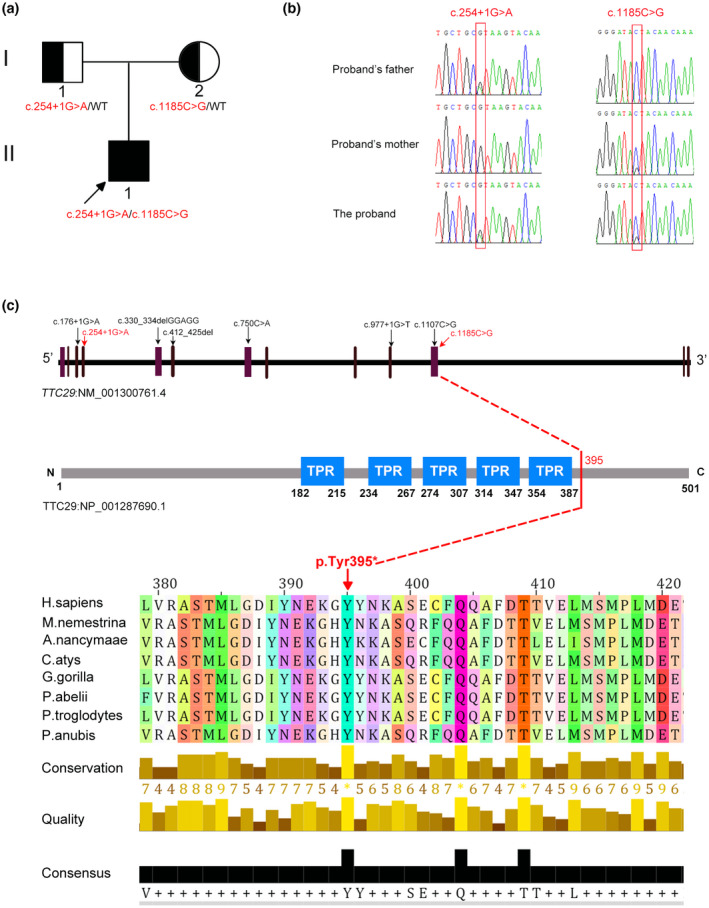
Identification of novel biallelic variants of TTC29 in an infertile male. (a) Family pedigree of the patient. The square represents the male, the circle represents the female, the black‐and‐white filled figure stands for the variation carrier, the black square represents the affected individual, and the arrow indicates the proband. (b) Sanger sequencing confirmed the mutations of NC_000004.12:g.146937593C>T (c.254+1G>A) and NM_001300761.4:c.1185C>G (NP_001287690.1:p.Tyr395*) in this MMAF family. Two red solid boxes denote the mutation positions. (c) TTC29 localization of variants in the genome, protein structure and multiple sequence alignment of the TTC29 protein for different species. The red arrow indicates the position of the TTC29 mutation in our study, and the black arrow indicates the mutations that have been reported in the previous studies.

To explore the genetic cause of MMAF in this study, whole‐exome sequencing was carried out on the affected patient. A nonsense variant, NM_001300761.4:c.1185C>G (NP_001287690.1:p.Tyr395*), and a splice‐site variant, NC_000004.12:g.146937593C>T (c.254+1G>A), in *TTC29* were identified, which had not been mentioned or had extremely frequency in the public databases, i.e., ExAC Browser, gnomAD, or the 1000 genome Project. The splicing variation was predicted to affect the splice site by SPIDEX and SpliceAI tools (Table 2). In addition, the biallelic mutations in *TTC29* were classified as “likely pathogenic” according to the ACMG guidelines. These two mutations were also not detected in our 500 fertile controls. Sanger sequencing was further applied to verify the mutations in the patient and his unaffected parents, which corresponds to the recessive inheritance nature of MMAF (Figure 2a,b). In addition, the site of NM_001300761.4:c.1185C>G variation is highly conserved among various species (Figure 2c). In summary, we speculated that the compound heterozygous mutations in *TTC29* accounted for the MMAF phenotype in the infertile patient.

**TABLE 2:** Variant analysis in the patient

	M1	M2
cDNA mutation^a^	c.1185C>G	c.254+1G>A
Protein changes	p.Tyr395*	–
Mutation type	Nonsense	Splicing
Genotype	Heterozygous	Heterozygous
Allele frequency		
in ExAC browser	0	0.000462
GnomAD	0	0.000190697
1000 Genomes Project	0	0.000599042
Function prediction		
dpsi_zscore^b^	–	−3.141
SpliceAI score^c^	–	0.78

Abbreviations: M1, mutation 1; M2, mutation 2.

^a^ NCBI reference sequence number of *TTC29* is NM_001300761.4 (https://www.ncbi.nlm.nih.gov/genbank/).

^b^ Absolute values of the score >2 are considered to be deleterious.

^c^ Scores >0.5 are suggested to affect splicing.

**FIGURE 3:**
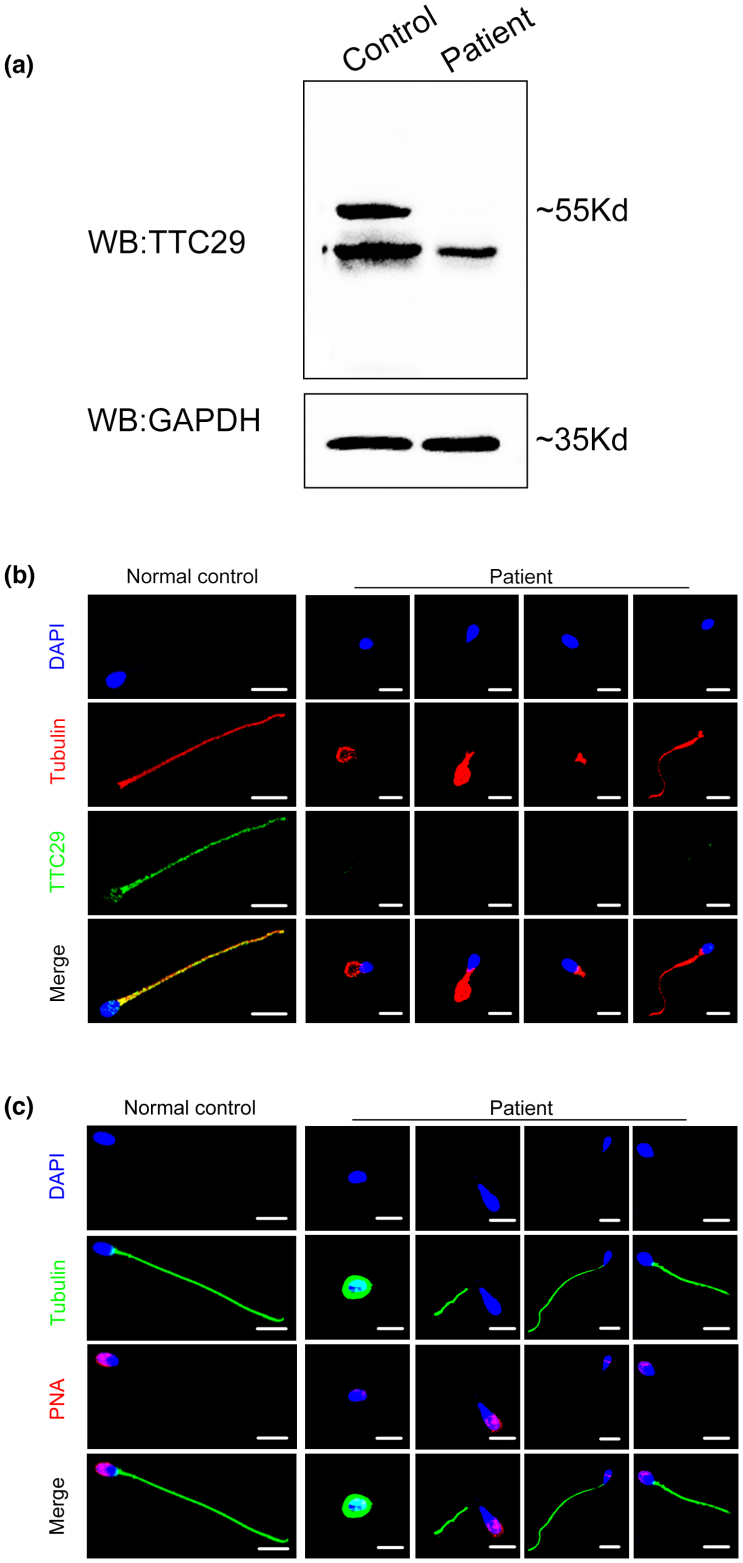
The impact of biallelic mutations in TTC29 on its expression. (a) The expression of TTC29 was absent in the patient's sperm by western blotting analysis. (b) The immunofluorescence staining reflected the absence of TTC29 expression of the patient's sperm compared to the normal control (blue, DAPI; red, αtublin; green, TTC29; scare bars, 5 μm). (c) PNA staining in the sperm cells revealed a defection of the acrosome structure in the patient compared to the normal individual (blue, DAPI; green, α‐tublin; red, PNA; scare bars, 5 μm).

To explore the negative effect of *TTC29* mutations on its expression, the western blotting results showed that TTC29 protein was absent in the sperm lysate from the patient harboring *TTC29* mutations compared to the normal control (Figure 3a). Notably, we performed immunofluorescence analysis with an anti‐TTC29 antibody on the sperm of the patient, and TTC29 staining was not detected in the sperm of the patient. Compared to the patient, TTC29 protein was detected along the sperm flagellum of the normal control (Figure 3b). Additionally, TTC29 staining was hardly detected in the patient's testicular tissue compared to the normal control (Figure 5a). These findings indicate that the mutations in *TTC29* in our study could induce the degradation of TTC29 protein and suggest that absent TTC29 expression in the sperm tail of the patient may impair the development of sperm flagellum, which further leads to the MMAF phenotype.

### 3.3 *TTC29* expression in human and mouse testicular tissues

**FIGURE 4:**
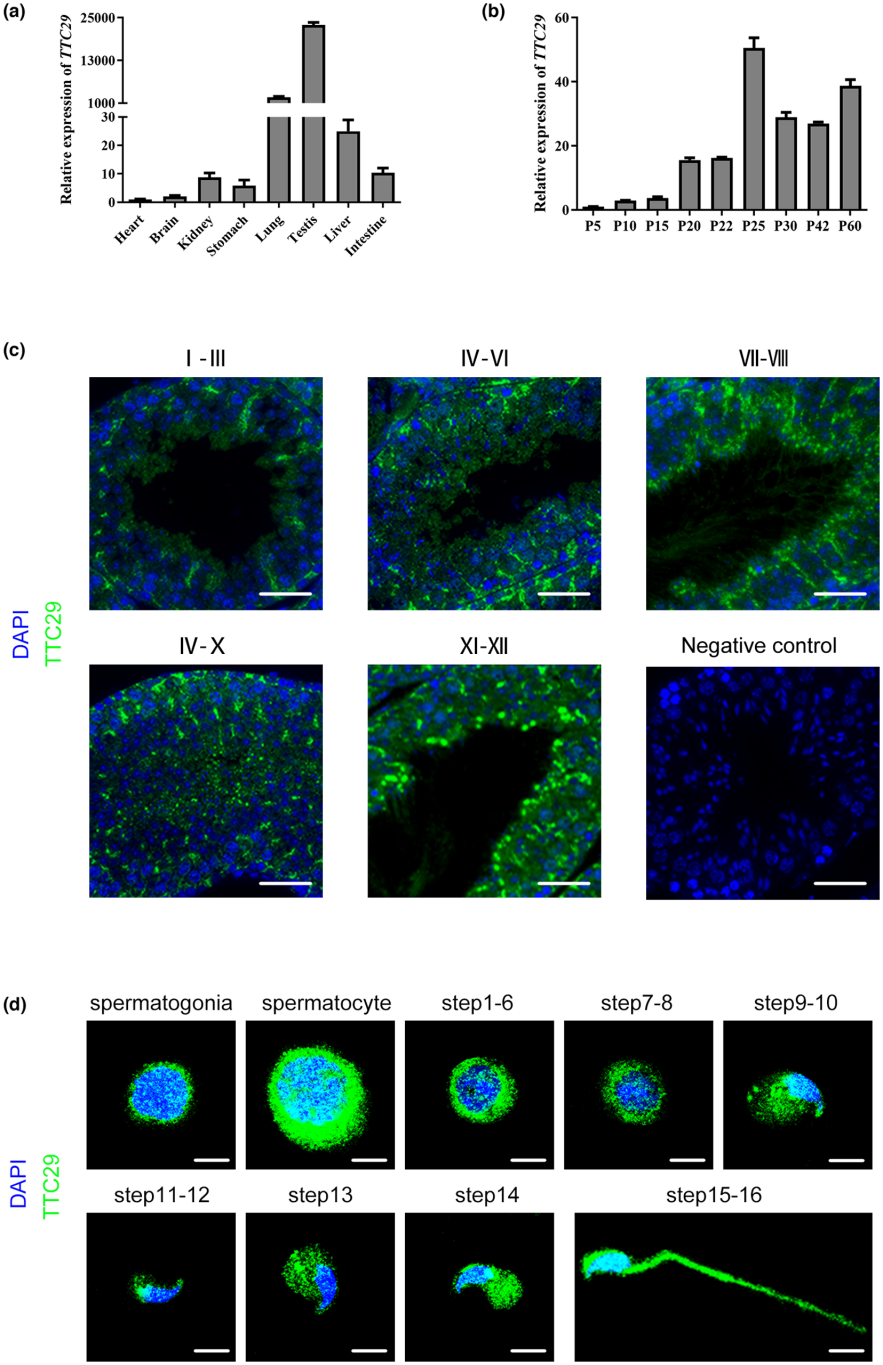
TTC29 expression in mouse tissues. (a) RT‐PCR revealed the expression of TTC29 in the different mouse tissues. The result was performed by a histogram based on the cycle threshold value. (b) RT‐PCR analysis revealed the expression of TTC29 in the different stage of mouse testes. Quantification of the RTPCR results by a histogram according to the cycle threshold value. (c) Immunofluorescence staining for various stages of mouse spermatogenesis using mouse testis sections. (green, TTC29; blue, DAPI; scale bars, 50 μm). (d) Immunofluorescence staining for different stages of mouse spermatogenic cells. TTC29 was dominantly localized in the cytoplasm of spermatogonia, spermatocyte, round spermatids, and the flagella of elongating or elongated spermatids. (green, TTC29; blue, DAPI; scale bars, 5 μm).

**FIGURE 5:**
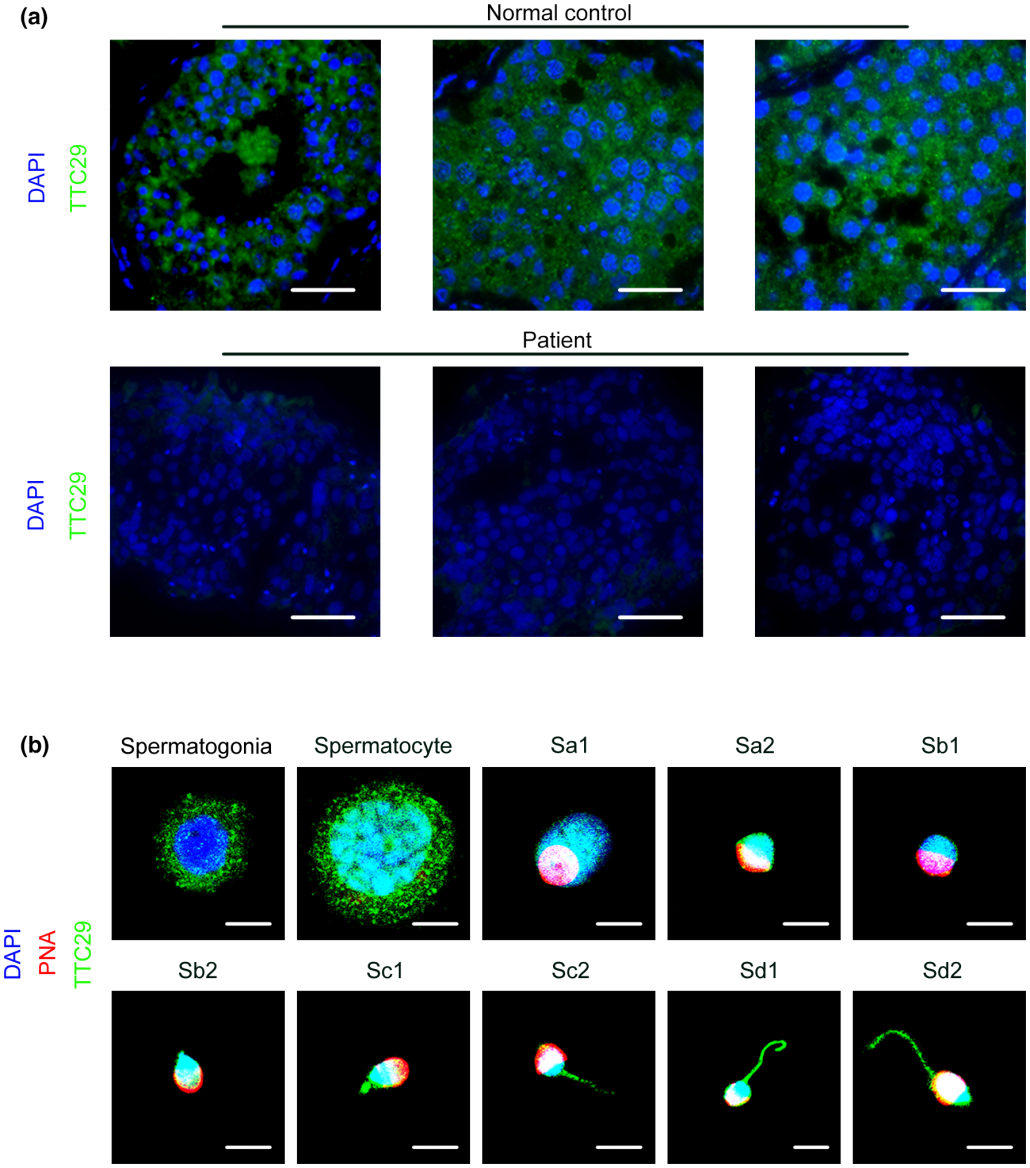
TTC29 expression in human testes. (a) Immunofluorescence staining showed the TTC29 protein in the patient's testicular tissues compared to the normal control (green, TTC29; blue, DAPI; scale bars, 50 μm). (b) Colocalization of TTC29 and PNA was observed in human early and late spermatids (green, TTC29; blue, DAPI; red, PNA; scale bars, 5 μm).

To further explore the important role of *TTC29* in male reproduction, various organs of adult mice, including the heart, brain, kidney, stomach, lung, testis, liver, and intestine were examined for *TTC29* expression by using real‐time PCR. The results showed that *TTC29* was predominantly expressed in mouse testes (Figure 4a). To investigate the expression of *TTC29* mRNA abundance in testicular tissue in mice of different postnatal days of age, we also used real‐time PCR to examine *TTC29* expression among testicular tissue at different periods. As the results indicated in Figure 4b, *TTC29* first appeared at postnatal Day 5, reached its peak at postnatal Day 25, and then sustained a stable expression level as far as postnatal Day 60, which was the latest timepoint. In addition, to determine the localization of *TTC29* in sections of mouse and human testes, immunofluorescence was used to stain testis sections with anti‐TTC29 antibody. Immunofluorescence staining of testis sections showed that *TTC29* was expressed in both mouse (Figure 4c) and human (Figure 5a) during spermatogenesis, and it was predominantly expressed in the cytoplasm of round and elongated spermatids from the sections of mouse and human testes. In addition, germ cells at different steps from mouse and human testicular tissue were sorted by the STA‐PUT velocity sedimentation method. Immunofluorescence staining showed that in mouse germ cells, *TTC29* is predominantly expressed in the cytoplasm of spermatogonia, spermatocyte, round spermatids (steps 1–8), early elongating spermatids (steps 9–12) and late elongating spermatids (steps 13–16) (Figure 4d). Furthermore, colocalization of *TTC29* and PNA showed that human *TTC29* was detected at the acrosomal and flagella regions of early and late spermatids (Figure 5b), which provided strong evidence that the loss‐of‐function mutations in *TTC29* could induce abnormal acrosomes. Overall, these results suggested that *TTC29* might be involved in the development of sperm flagellum.

### 3.4 Outcomes of ICSI in the patient harboring *TTC29* mutations

To achieve pregnancy, the couple accepted ICSI in our center after obtaining written informed consent. The patient's sperm with relatively normal morphology were selected for ICSI treatment. The patient's wife had a regular menstrual cycle and normal endocrine indices. She underwent the long gonadotrophin‐releasing hormone (GnRH) agonist protocol in the first cycle (Table 3). Nine oocytes were retrieved after GnRH treatment. Then, five mature oocytes (metaphase II) were microinjected, and three oocytes were ultimately fertilized (2PN/injected oocytes = 60%). Although all three embryos reached the cleavage stage, none of them continued to develop. In the second cycle, the couple went for the antagonist protocol. We retrieved four metaphase II oocytes and injected them; three were ultimately fertilized. Regrettably, they failed to develop after reaching the available D3 stage.

**TABLE 3:** Clinical features of the patient's spouse with ICSI treatment

	Spouse of patient
Age (years)	36
Length of primary infertility history (years)	6
BMI	20.2
Basal hormones	
FSH (IU/L)	12.7
LH (IU/L)	5.8
E2 (pg/ml)	36.9
Prog (ng/ml)	0.43
Cycle 1	
Protocol	Long
E2 level on the trigger day (pg/ml)	1327.0
No. of follicles ≥14 mm on the trigger day	9
No. of follicles ≥18 mm on the trigger day	3
No. of oocytes retrieved	9
ICSI progress	
Oocytes injected	5
Fertilization rate (%)	60 (3/5)
Cleavage rate (%)	100 (5/5)
Available D3 embryos	3
Cycle 2	
Protocol	Antagonist
E2 level on the trigger day (pg/ml)	1285.1
No. of follicles ≥14 mm on the trigger day	6
No. of follicles ≥18 mm on the trigger day	2
No. of oocytes retrieved	8
ICSI progress	
Oocytes injected	4
Fertilization rate (%)	75 (3/4)
Cleavage rate (%)	100 (4/4)
Available D3 embryos	3

## 4 DISCUSSION

We discovered two loss‐of‐function mutations, NM_001300761.4:c.1185C>G and NC_000004.12:g.146937593C>T (c.254+1G>A), in *TTC29*, which were unexplored in previous studies. In addition to the typical spermatozoa MMAF morphology from the patient, we intriguingly detected abnormal morphology of the patient's sperm heads, presenting the various head shapes and the reduction or absence of acrosomes. We also explored the expression of *TTC29* in multiple germ cells of humans and mice. Our evidence strongly supports that the loss‐of‐function mutations in *TTC29* are associated with MMAF, thus resulting in male infertility.

The TTC29 protein consists of five tetratricopeptide repeat (TPR) domains, which are 34 amino acid repeats present in various proteins, forming alpha helixes and behaving as scaffolds for protein–protein interactions and assembly of multiprotein complexes involved in many cellular processes (Allan & Ratajczak, 2011; Blatch & Lässle, 1999; Perez‐Riba & Itzhaki, 2019; Zeytuni & Zarivach, 2012). Previous studies also indicated that TPR family proteins play an important role in cilia‐ and flagella‐associated functions. Cilia and flagella are microtubule‐based organelles, and their assembly requires a motile process known as intraflagellar transport (IFT). IFT has a bidirectional protein transport system inside cilia. IFT complex B plays a major role in the assembly and maintenance of cilia and flagella, regulating the anterograde transport of ciliary components from the cell body to the tip of cilia, while IFT complex A regulates retrograde transport, sending the products of turnover back to the cell body from cilia (Hou et al., 2007; Ishikawa & Marshall, 2017; Rosenbaum, 2002; Scholey, 2003). Work from Mei‐I Chung et al. demonstrated that partial knockdown of *TTC29* significantly decreased the mean rate of anterograde IFT, which is consistent with the bioinformatic linkage of *TTC29* to components of the anterograde IFT complex B, indicating that *TTC29* is part of IFT complex B (Chung et al., 2014). In our study, we identified novel biallelic mutations in *TTC29*, including a nonsense variant, NM_001300761.4:c.1185C>G (NP_001287690.1:p.Tyr395*), and a splice‐site variant, NC_000004.12:g.146937593C>T (c.254+1G>A). The anti‐TTC29 antibody recognition site is “MTRPKLTALARQKLPCSSRKIPRSQLIKEKDDIDHYLEVNFKGLSKEEVAAYRNSYKKNICVDMLRDGYHKSFTELFALMERWD” and this sequence of amino acid is from 35 to 118. In our study, the amino acid site corresponding to the nonsense mutation (NM_001300761.4:c.1185C>G) site is 395; another amino acid site corresponding to the splicing mutation (NC_000004.12:g.146937593C>T) site is about 85. The protein sequence before the two sites can be recognized by the antibody. Therefore, if the truncated protein caused by the two biallelic mutations in *TTC29* in our study does not degrade, the antibody could recognize it and the TTC29 band can be detected. However, TTC29 protein was absent in the sperm lysate from the patient according to the western blotting results in our study, we thus speculated that these two mutations in *TTC29* led to protein degradation, and further induced MMAF in the patient. Our findings are consistent with previous results, which provide strong evidence that *TTC29* participates in the composition of IFT complex B and plays an important role in the flagellum assembly process. Collectively, the study of loss‐of‐function mutations in *TTC29* in the previous study and in our case jointly proved that the *TTC29* variants were genetic causes of MMAF‐associated asthenoteratospermia and provided a richer clinical basis for the deep functional exploration and mechanism research of *TTC29*.

ICSI is a widespread technique used to treat MMAF‐associated individuals. However, previous studies indicated that MMAF cases caused by different mutations exhibit different prognoses following ICSI. For example, patients with mutations in *DNAH1*, *DNAH8*, *TTC29*, *CFAP44*, and *CFAP43* experienced favorable ICSI outcomes, while mutations in *DNAH17*, *CEP135*, and *FSIP2* experienced disappointing outcomes (Liu et al., 2020; Sha et al., 2019; Wambergue et al., 2016). In our study, ICSI treatment had a poor outcome and the finding differs from previously reported results, which showed good prognosis for ICSI. From relevant sequencing results of the patient, there were no any other pathogenic genes related to fertilization and embryonic development (Table S1). Therefore, the failure of patient's ICSI treatment may be caused by other factors. For example, additional female infertility risk factors should not be excluded.

In conclusion, although the specific mechanisms underlying mutations in *TTC29* that cause MMAF require further exploration, our genetic and functional analyses in an affected patient suggest that biallelic variants of *TTC29* induce MMAF‐associated male infertility. Furthermore, our work might provide more detailed information on the pathogenesis of male infertility induced by *TTC29* mutations. In addition, our findings expand the mutational spectrum of *TTC29*. Overall, this study provides new, important knowledge for genetic counselors and clinicians to further understand the genetic causes of asthenoteratospermia and male infertility and help them establish effective interventions or individualized treatment plans.

## AUTHOR CONTRIBUTIONS

H.L. and Y.S. designed and supervised the study experiments. H.L., Y.S., S.D., and Y.L. collected data and conducted the clinical evaluations. M.L. and Y.Y. performed TEM and SEM. The first draft of the manuscript was written by S.D. and all authors commented on previous versions of the manuscript. S.D., M.L., Y.Y., and Y.L. performed immunofluorescence staining. All authors read and approved the final manuscript.

## FUNDING INFORMATION

This work was supported by the West China Second University Hospital of Sichuan University (no. KS369).

## CONFLICT OF INTEREST

This manuscript has not been published or presented elsewhere in part or in entirety and is not under consideration by another journal. All study participants provided informed consent, and the study design was approved by the appropriate ethics review board. The authors declare no conflict of interest.

## ETHICS STATEMENT

The studies involving human participants were approved by the Ethical Review Board of West China Second University Hospital, Sichuan University. Informed consent was obtained from all individual participants included in the study.

## DATA AVALABILITY STATEMENT

The data analyzed in this study is subject to the following licenses/restrictions: the datasets for this article are not publicly available because of privacy concerns. Requests to access these datasets should be directed to YS, yingcaishen01@163.com.

## Supporting information


Figure S1

Table S1
