# Supplementary material for: Novel biallelic mutations in TTC29 cause asthenoteratospermia and male infertility
Source: Mol Genet Genomic Med. 2022 Nov 8;10(12):e2078. doi: 10.1002/mgg3.2078 (PMC9747556; doi:10.1002/mgg3.2078)
Supplement: Supplementary file 1 — Figure S1 [file MGG3-10-e2078-s001.pdf]

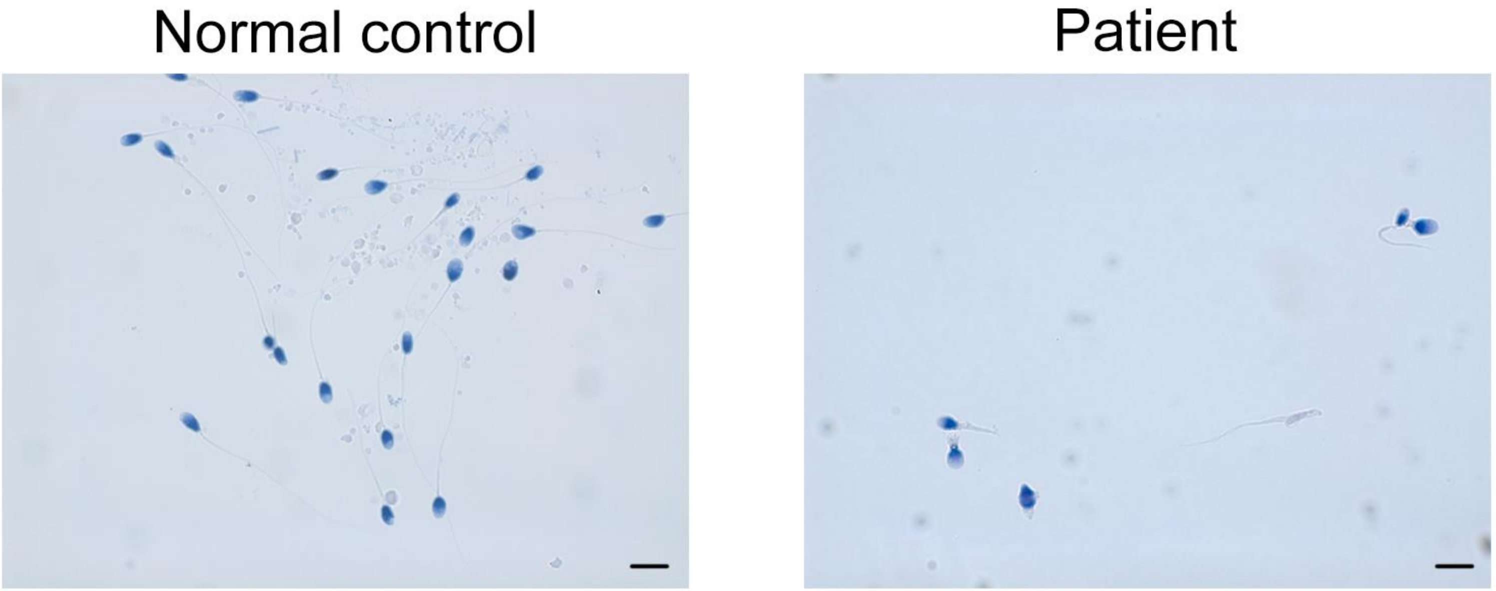

Figure S1. The aberrant morphology of spermatozoa in the patient. The patient's sperm showed short, coiled, and absent flagella in a low magnification image compared to the normal control (scale bars, 5 μm).
